# Association Between DMFT Index and Sinonasal Anatomical Variations in Children: A Retrospective Cone-Beam Computed Tomography Study

**DOI:** 10.3390/children13081095

**Published:** 2026-08-18

**Authors:** Mehmet Veysel Kotanlı, Mehmet Emin Dogan, Şeyda Alkan, Sedef Kotanlı

**Affiliations:** 1 Department of Pediatric Dentistry, Faculty of Dentistry, Harran University, Sanliurfa 63300, Türkiye; 2 Department of Dentomaxillofacial Radiology, Faculty of Dentistry, Harran University, Sanliurfa 63300, Türkiye

**Keywords:** child, cone-beam computed tomography, dental caries, DMF index, nasal septum, paranasal sinuses

## Abstract

**Highlights:**

**What are the main findings?**

- More severe nasal septal deviation was associated with higher DMFT (decayed, missing, and filled teeth) scores in children.
- Concha bullosa and Haller cells were not significantly associated with DMFT (decayed, missing, and filled teeth) scores.
- Lamellar concha bullosa was the most frequently observed concha bullosa subtype on both sides.

**What are the implications of the main findings?**

- Cone-beam computed tomography may provide additional information about sinonasal anatomical variations when scans are already available for pediatric dental indications.
- Severe nasal septal deviation may be considered a radiological feature associated with higher DMFT (decayed, missing, and filled teeth) scores, but not an independent clinical predictor of caries experience.
- The potential relevance of this association for multidisciplinary pediatric dental and otorhinolaryngological assessment should be further evaluated in prospective studies.

**Abstract:**

Background/Objectives: This retrospective study aimed to evaluate the association between decayed, missing, and filled teeth (DMFT) scores and sinonasal anatomical variations, including Haller cells, concha bullosa, and the direction and severity of nasal septal deviation in children. Methods: Cone-beam computed tomography (CBCT) images of 167 patients aged 6–17 years were retrospectively analyzed. The direction and severity of nasal septal deviation, presence of Haller cells, presence and subtypes of concha bullosa, and DMFT index were evaluated. Nasal septal deviation was categorized as mild (<9°), moderate (9–15°), or severe (≥15°). Concha bullosa was classified as lamellar, bulbous, or extensive. DMFT scores were assessed radiologically using reconstructed panoramic images, without intraoral clinical examination data. The DMFT index was calculated only for permanent teeth. Results: Among the 167 patients, 87 were male and 80 were female. Mild nasal septal deviation was the most common type, observed in 63.5% of cases. Lamellar concha bullosa was the most frequently detected subtype on both sides. No statistically significant association was found between concha bullosa or Haller cells and DMFT scores (*p* > 0.05). However, a significant positive ordered trend was observed between increasing nasal septal deviation severity and DMFT scores (*p* = 0.004). Conclusions: Increased nasal septal deviation severity was associated with higher DMFT scores in children. However, because of the retrospective design and radiological assessment of DMFT, this association should be interpreted as a potential relationship rather than a causal effect.

## 1. Introduction

The nasal cavities and paranasal sinuses are among the anatomical structures that most frequently display variations in the human body [1]. Among these, nasal septal deviation (NSD), concha bullosa (CB), and Haller cells (HC) are particularly common, and these variations may contribute to the narrowing of the osteomeatal complex and sinonasal drainage pathways, thereby compromising sinus ventilation and increasing nasal airflow resistance [2,3].

NSD is a prevalent anatomical variation characterized by deviation of the nasal septum from the midline, resulting in possible narrowing or obstruction of the nasal cavity. It can present at different levels of severity, classified as mild, moderate, or severe [4]. Depending on its severity and anatomical localization, NSD may lead to significantly impaired nasal airflow, necessitating a transition to compensatory mouth breathing [5].

CB represents an anatomical variation defined by the presence of partially or completely pneumatized air cells within the middle turbinate. It is one of the most frequently encountered anomalies of the osteomeatal region, although its precise etiology remains unclear. The clinical significance of CB lies primarily in its potential to narrow the nasal valve and disrupt the functional integrity of the osteomeatal complex, potentially increasing nasal airway resistance [6].

HC refers to infraorbital ethmoidal pneumatization [7]. Its clinical relevance derives from the potential to exert obstructive effects at the posterior portion of the ethmoidal infundibulum and the maxillary sinus ostium. Anatomically, HCs located in this region may compromise the drainage function of the osteomeatal complex, thereby predisposing patients to chronic rhinosinusitis and persistent nasal obstruction, which may necessitate chronic mouth breathing [7,8].

Anatomical variations such as NSD, CB, and HC may contribute to sinonasal airflow limitation or obstruction. NSD and CB have been described as potential factors affecting the nasal respiratory complex, while HC may have a possible obstructive role by narrowing the ethmoidal infundibulum or maxillary ostium [6,8]. Impaired nasal breathing or nasal obstruction may promote a shift from nasal to oral breathing [9,10]. Mouth breathing may alter the oral environment by increasing oral dehydration and affecting salivary and microbial homeostasis. These changes may reduce intraoral pH and favor the growth of cariogenic microorganisms, thereby creating conditions that could contribute to dental caries development [9].

The DMFT index (Decayed, Missing, and Filled Teeth) is globally recognized as one of the most important indices for assessing oral and dental health status [11]. However, DMFT scores are multifactorial and may be influenced by several behavioral, environmental, and socioeconomic factors, including oral hygiene habits, dietary patterns, sugar consumption, fluoride exposure, parental factors, and access to dental care [12,13,14,15]. Therefore, evaluating the possible relationship between upper airway anatomical variations and DMFT scores may help identify additional factors associated with caries experience, although conventional caries risk factors should also be considered.

Recent pediatric imaging studies have emphasized that the paranasal sinuses and sinonasal structures continue to develop throughout childhood and adolescence, with their morphology varying according to age [16,17]. CBCT-based studies involving children and patients of developmental age have also reported that sinonasal anatomical variations, such as NSD, CB, and HC, are commonly detected as incidental findings during dental imaging [18,19]. Previous studies have investigated the relationship between the DMFT index and various conditions, such as obstructive sleep apnea [20], salivary characteristics [21], mouth breathing [22], adenotonsillar hypertrophy [23] and allergic rhinitis [24]. However, these studies have primarily focused on soft tissue obstructions, respiratory conditions, salivary factors, or systemic factors, leaving the relationship between specific sinonasal anatomical variations and dental caries experience insufficiently investigated. To the best of our knowledge, the association of NSD severity, CB subtypes, and HC presence with the DMFT index has not yet been specifically examined in children. We hypothesized that higher NSD severity would be associated with increased DMFT scores, whereas the presence of CB and HC would not show a direct significant association with DMFT scores. Accordingly, the present study aimed to evaluate the association between DMFT scores and HC presence, CB subtypes, and the direction and severity of NSD in pediatric CBCT images. By addressing this gap, the study may provide valuable interdisciplinary insight into pediatric dental and sinonasal assessment, thereby supporting the development of multidisciplinary diagnostic and therapeutic approaches involving otorhinolaryngology and pediatric dentistry.

## 2. Materials and Methods

This study was conducted with the approval of the Ethics Committee of Harran University Faculty of Dentistry (Date: 14 July 2025, Decision No: 23/13/55). All procedures were carried out in accordance with ethical standards and the principles of the Declaration of Helsinki. No CBCT scan was performed specifically for the purpose of this study; all images had been acquired previously for diagnostic purposes. In this retrospective study, cone-beam computed tomography (CBCT) images obtained from pediatric patients who presented to the Department of Oral, Dental and Maxillofacial Radiology, Faculty of Dentistry, Harran University, between 2021 and 2025 for various reasons were evaluated in terms of the severity and direction of NSD, the presence of HC, the presence and subtypes of CB, and the DMFT index.

A sample size calculation was performed using G*Power software, version 3.1.9.7. For the multiple linear regression analysis, the “Linear multiple regression: Fixed model, R^2^ increase” option was selected under the F tests family. The calculation was performed using a small-to-moderate effect size (f^2^ = 0.05), an alpha error probability of 0.05, a statistical power of 0.80, one tested predictor, and three total predictors. The minimum required sample size was calculated as 159 patients. Since the present study included 167 patients, the sample size was considered sufficient for the planned regression analysis.

CBCT images of 167 patients aged 6 to 17 years were included. Inclusion criteria required the absence of defects, lesions, or pathologies in the maxillofacial region, no history of jaw fractures or related surgical procedures, and no systemic or genetic diseases affecting bone metabolism. Conversely, low-resolution images or those containing significant patient- or device-related artifacts that hindered anatomical evaluation were excluded from the study.

CBCT scans were acquired using a Castellini X-Radius Trio Plus device (CEFLA S.C., Imola, Italy) with exposure parameters of 90 kVp and 16 mAs. Images were obtained with a 0.3 mm voxel size and a 13 × 16 cm or 13 × 10 cm field of view (FOV), depending on the clinical indication. All datasets were reconstructed at a 0.3 mm slice thickness using IRYS Viewer 15.1 software for standardized anatomical assessment.

**Figure 1:**
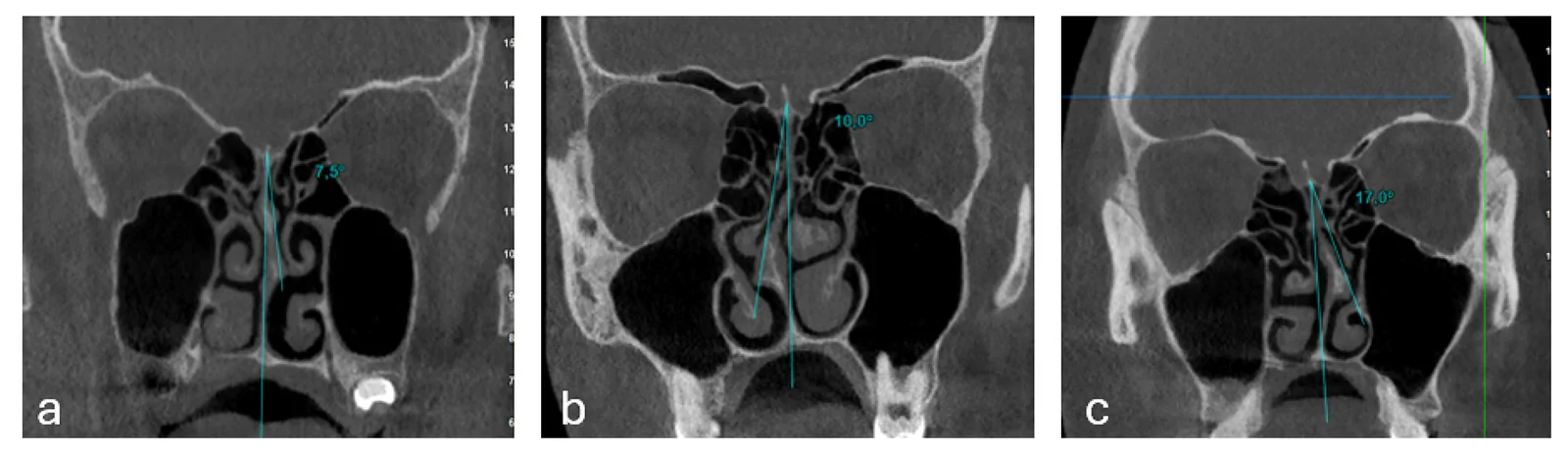
Representative coronal cone-beam computed tomography (CBCT) images showing nasal septal deviation (NSD) types: (**a**) Type I (<9°, mild), (**b**) Type II (9–15°, moderate), and (**c**) Type III (≥15°, severe).

**Figure 2:**
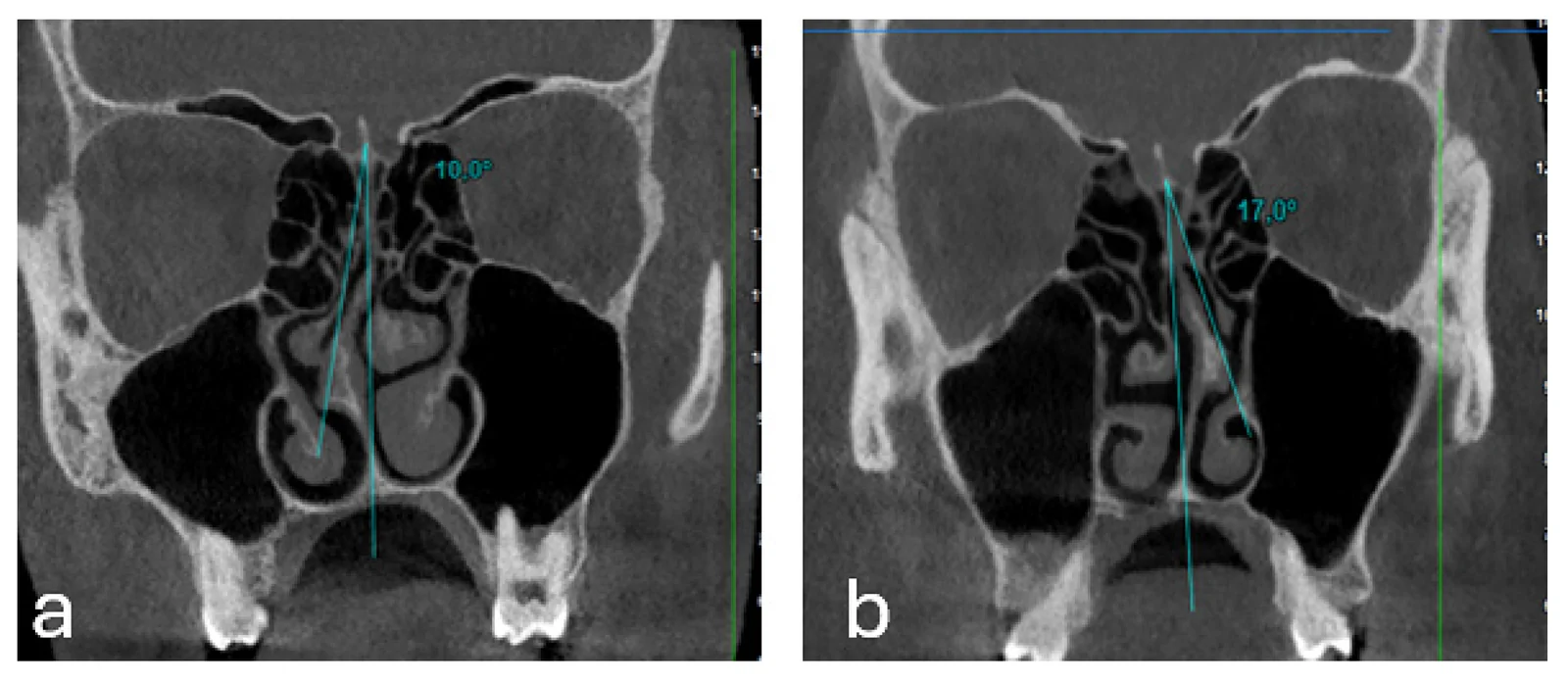
Representative coronal cone-beam computed tomography (CBCT) images showing nasal septal deviation (NSD) directions: (**a**) right-sided NSD and (**b**) left-sided NSD.

NSD was graded using coronal CBCT images. The deviation angle was measured between a vertical reference line extending from the premaxilla to the crista galli and a line directed toward the point of maximum septal convexity. Following the classification by Elahi et al. [25], deviations were categorized based on the measured angle as Type I (mild, <9°), Type II (moderate, 9–15°), and Type III (severe, ≥15°) (Figure 1). Additionally, the direction of the deviation (right or left) was recorded for each patient (Figure 2).

**Figure 3:**
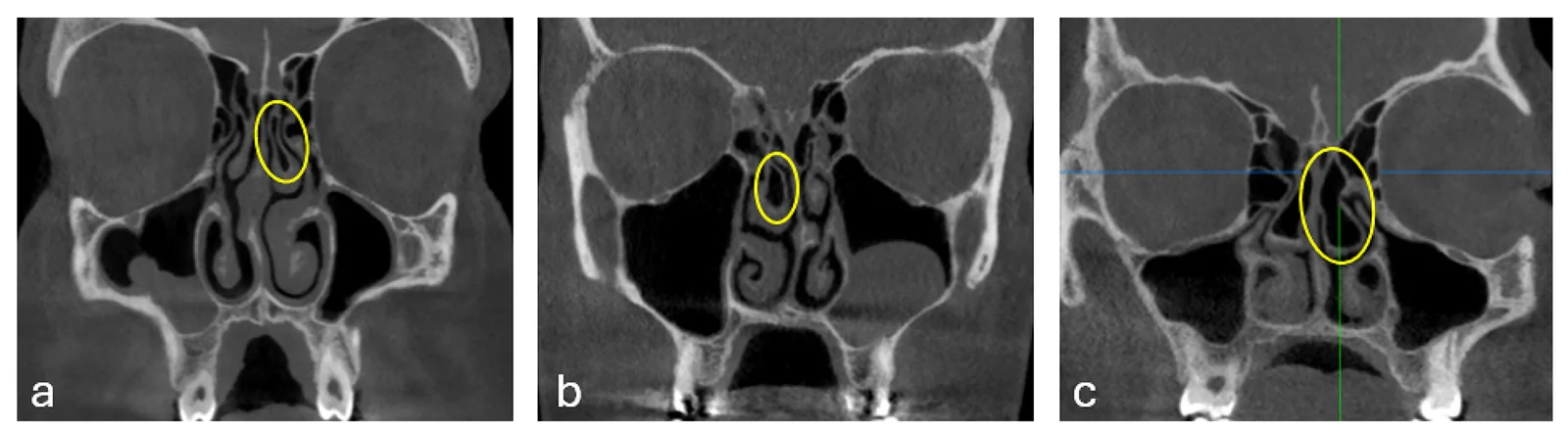
Representative coronal cone-beam computed tomography (CBCT) images showing concha bullosa (CB) types: (**a**) lamellar, (**b**) bulbous, and (**c**) extensive.

The classification of CB was based on the criteria established by Bolger et al. [26]. The middle turbinate pneumatization was evaluated in three distinct categories according to its extent: lamellar (pneumatization of the vertical lamella), bulbous (pneumatization of the distal bulbous portion), and extensive (pneumatization of both the lamella and the bulbous portion) (Figure 3). Assessments were performed using both coronal and axial CBCT planes to ensure accurate anatomical identification.

**Figure 4:**
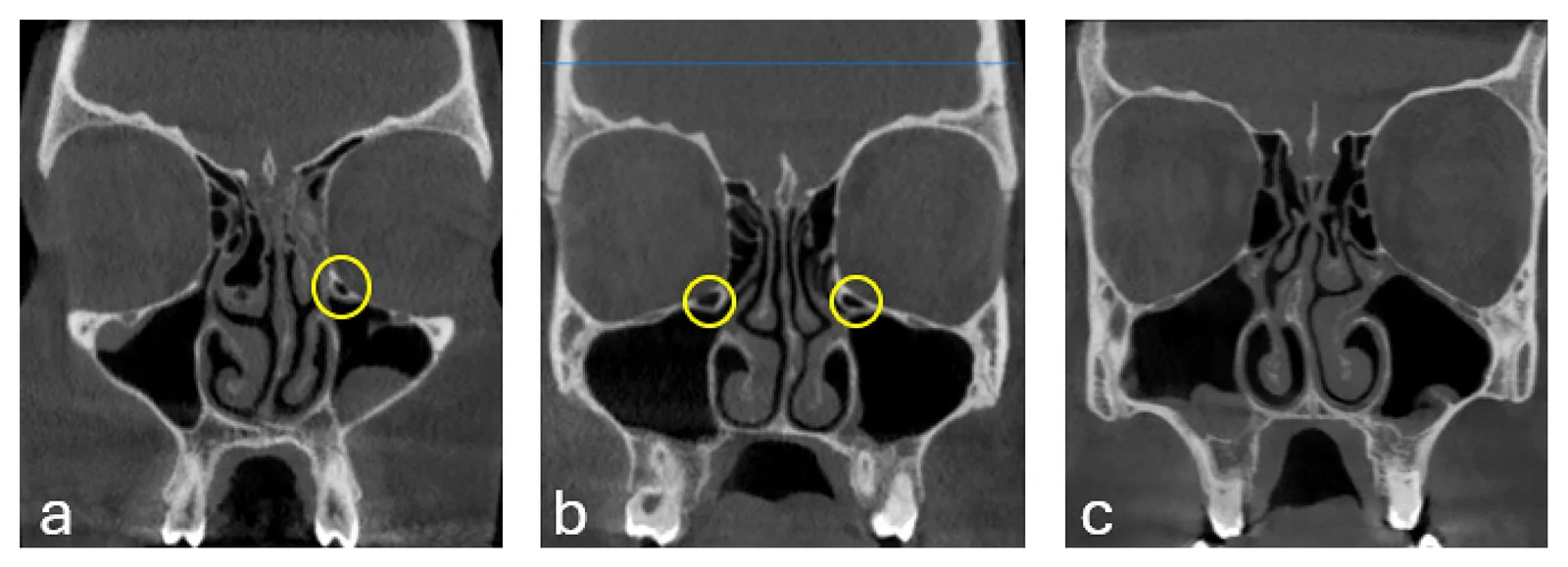
Representative coronal cone-beam computed tomography (CBCT) images showing Haller cells (HC): (**a**) unilateral HC on the left side, (**b**) bilateral HC on both sides, and (**c**) absence of HC.

The presence of HC was evaluated based on the criteria defined by Mathew et al. [27]. These structures were identified as infraorbital ethmoidal air cells localized at the roof of the maxillary sinus, superior to the sinus ostium, and medially to the infraorbital canal. Special care was taken to differentiate HCs from the infraorbital canal and the bulla ethmoidalis. The presence or absence of these morphological variations was recorded for each side (Figure 4).

For dental evaluation, three-dimensional volumetric data were processed to generate high-quality two-dimensional panoramic reconstructions. This was achieved by creating a reference line along the dental arch on cross-sectional slices. The DMFT index (Decayed, Missing, and Filled Teeth) was assessed radiologically by evaluating these reconstructed images for evidence of cavitated caries, missing teeth due to pathology, and radiopaque restorations. Due to the retrospective nature of the study, intraoral clinical examination data were not utilized. The DMFT index was calculated only for permanent teeth. Primary teeth and teeth lost due to physiological exfoliation were not included in the index. For children in the mixed dentition period, including 6-year-old patients, the DMFT score was calculated only according to the erupted or radiographically visible permanent teeth. Therefore, in younger patients with a limited number of permanent teeth, the DMFT value represented permanent dentition caries experience rather than total oral caries experience.

Before the main assessment, the observers reviewed a set of representative CBCT images together to standardize the evaluation criteria for NSD measurement, CB classification, HC detection, and radiological DMFT assessment. All radiological assessments were then performed by two observers experienced in oral and maxillofacial radiology. Inter-observer agreement was calculated for the initial evaluations. To assess intra-observer reliability, 20% of the images were re-evaluated after a two-week interval. Agreement was calculated using the intraclass correlation coefficient for continuous measurements and Cohen’s kappa coefficient for categorical variables.

All statistical analyses were performed using SPSS for Windows, version 23.0 (IBM Corp., Armonk, NY, USA). Descriptive statistics were presented as mean ± standard deviation, median (minimum–maximum), and frequencies. The normality of data distribution was assessed with the Kolmogorov–Smirnov test. For comparisons between two independent groups that did not show normal distribution, the Mann–Whitney U test was applied. To evaluate the ordered directional relationship of DMFT scores across the three levels of NSD severity (Type 1, 2, and 3), the Jonckheere-Terpstra test was employed. Pearson’s Chi-square test was employed for the analysis of categorical variables. Multiple linear regression analysis was performed to identify factors associated with DMFT scores. Before linear regression analysis, model assumptions, including residual normality, homoscedasticity, and multicollinearity, were evaluated. In all analyses, a *p*-value < 0.05 was considered statistically significant.

## 3. Results

CBCT images of 167 patients aged between 6 and 17 years were examined. Among the patients, 80 (47.9%) were female and 87 (52.1%) were male, with a mean age calculated as 12.43 ± 2.77 years.

The reliability analysis demonstrated excellent agreement for continuous measurements (ICC > 0.90) and almost perfect agreement for categorical assessments (Cohen’s κ = 0.89).

When the distribution of NSD direction was evaluated, deviation to the left side was detected in 83 patients (49.7%), whereas deviation to the right side was detected in 84 patients (50.3%). Regarding the types of deviation, mild deviation (Type I) was identified in 106 patients (63.5%), moderate deviation (Type II) in 50 patients (29.9%), and severe deviation (Type III) in 11 patients (6.6%) (Table 1).

Regarding accessory anatomical variations, the prevalence of CB was determined as 59.9% on the right side and 55.7% on the left side, with the lamellar type being the most common on both sides (Right: 28.1%; Left: 25.7%). Furthermore, the presence of HC was identified at a rate of 28.1% on the right and 29.9% on the left. (Table 1).

Table 2 presents the relationship between NSD severity and direction and the presence of CB and HC on the right and left sides. On the right side, CB was observed in 58.5% of patients with Type I NSD, 62.0% of those with Type II, and 63.6% of those with Type III. There was no statistically significant association between NSD severity and the presence of CB on the right side (*p* = 0.885). Similarly, on the left side, although the frequency of CB varied across NSD types (Type I: 56.6%, Type II: 50.0%, Type III: 72.7%), this difference was not statistically significant (*p* = 0.370).

Likewise, the presence of HC was not significantly associated with NSD severity on either the right (*p* = 0.295) or left side (*p* = 0.623). In terms of NSD direction, among patients with right-sided CB, 64.3% had right-sided NSD and 55.4% had left-sided NSD, with no statistically significant difference (*p* = 0.243). On the left side, CB was present in 52.4% of patients with right-sided NSD and 59.0% of those with left-sided NSD (*p* = 0.387). Regarding HC, on the right side, 25.0% of patients with right-sided NSD and 31.3% of those with left-sided NSD had HC (*p* = 0.363). On the left side, HC was present in 32.1% of patients with right-sided NSD and 27.7% of those with left-sided NSD (*p* = 0.532). Overall, no significant associations were found between NSD direction and the presence of CB or HC.

The relationship between the presence of CB and HC and DMFT scores is presented in Table 3. Although mean DMFT values were slightly higher in patients with right-sided CB (2.78 ± 2.68) compared to those without (2.45 ± 3.22), this difference was not statistically significant (*p* = 0.079). Similarly, no significant difference in DMFT scores was observed between patients with and without left-sided CB (*p* = 0.282). With respect to HC, DMFT scores did not differ significantly according to their presence on either the right (*p* = 0.411) or left side (*p* = 0.308). Overall, the presence of CB or HC was not significantly associated with DMFT scores (*p* > 0.05 for all comparisons).

The relationship between NSD severity and DMFT scores is presented in Table 4. A significant positive ordered trend was identified between NSD severity levels and DMFT scores (*p* = 0.004). The Jonckheere-Terpstra test indicated that DMFT scores increased significantly across increasing NSD severity levels from Type I to Type III (Table 4).

The results of the multiple linear regression analysis conducted to determine the factors affecting the DMFT (Decayed, Missing, and Filled Teeth) index are presented in Table 5. The analysis revealed that the established model was statistically significant (*F* = 9.517; *p* < 0.001) and accounted for 14.9% of the variance in the DMFT scores (*R^2^* = 0.149).

When the independent variables in the model were examined, age (β = 0.308, *p* = 0.001) and nasal septal deviation (NSD) severity (β = 0.206, *p* = 0.005) were found to have a significant and positive predictive effect on the DMFT index. Accordingly, DMFT scores increased with age and greater NSD severity. On the other hand, the sex variable was found to have no statistically significant effect on the DMFT index (β = −0.026, *p* = 0.718).

## 4. Discussion

Various imaging modalities are employed for paranasal sinus evaluation, ranging from conventional radiography (Waters, Caldwell, lateral cephalometric, and submentovertex views) to advanced techniques like computed tomography (CT) and magnetic resonance imaging (MRI) [28,29]. Although conventional two-dimensional radiographs remain widely used because of their accessibility, lower cost, and relatively low radiation exposure, their diagnostic value may be limited by the lack of three-dimensional information and the superimposition of anatomical structures [30]. MRI may provide useful information for evaluating mucosal and soft-tissue changes in the paranasal sinuses; however, recent evidence suggests that its performance may be limited for assessing ostiomeatal obstruction and sinonasal anatomical variants that require detailed visualization of bony sinonasal structures [31].

Although CT is considered the gold standard for visualizing opacified sinuses and intricate bone anatomy, its clinical use is often restricted by high radiation doses, higher costs, and challenges in patients with claustrophobia [29,32]. In contrast, CBCT provides high-resolution three-dimensional imaging with a single rotation at a significantly lower radiation dose and cost compared to medical CT [32,33]. Therefore, CBCT was preferred in this study to accurately evaluate NSD severity, CB types, and the presence of HC, while also providing high-quality reconstructed images for DMFT assessment.

Anatomical variations in the nasal and paranasal structures may affect sinonasal airflow and, when nasal breathing is impaired, compensatory mouth breathing may occur. Previous studies have suggested that mouth-breathing-related changes may alter the oral environment by affecting salivary protection, oral microflora, and self-cleansing mechanisms, which may contribute to caries susceptibility [9,22,23]. In the present study, this pathway may help explain the observed relationship between NSD severity and DMFT scores; however, mouth-breathing status, nasal airflow, salivary flow, oral pH, and oral microbiota were not directly evaluated.

Existing literature offers limited studies investigating the prevalence of Nasal Septal Deviation (NSD) and its potential associations with osteomeatal complex variations such as HC and CB [5,34]. Furthermore, to the best of our knowledge, this is the first study to concurrently evaluate NSD severity, CB subtypes, HC presence, and the DMFT index. Consequently, we believe our findings provide a significant contribution by bridging the gap between sinonasal anatomical variations and dental health outcomes.

Various classification systems for NSD have been proposed, focusing on different morphological features. Mladina [35] categorized NSD into seven types, while Vidigal et al. [36] and Rohrich et al. [37] classified deviations based on their relationship with the inferior turbinate or the degree of concavity, respectively. However, many of these systems lack a quantitative angular component. To address this limitation, we utilized the classification proposed by Elahi et al. [25], which provides a more objective and reproducible quantitative approach by measuring the precise angle of deviation.

Our results regarding the distribution of NSD severity are consistent with the findings of Şahin et al. [38], where the frequency of NSD followed a decreasing pattern from Type I to Type III. In our study, Type I was the most prevalent, followed by Type II and Type III. In contrast, another radiological study involving an adult population reported Type II as the most common form [39]. This discrepancy in prevalence may be attributed to differences in the age ranges of the study populations, as our study specifically focused on pediatric and adolescent patients (aged 6–17 years).

Regarding the direction of deviation, Mundra et al. [40] reported a higher frequency of left-sided NSD (59.01%). Similarly, another study reported that NSD was more frequently seen on the left side (left: 43.9%/right: 36.4%) [41]. In the present study, right- and left-sided NSD were observed at nearly equal frequencies. Although this result differs from previous studies reporting a predominance of left-sided deviation [6,42], it is consistent with a recent CBCT-based study reporting similar frequencies of right- and left-sided NSD [43].

Kar et al. [44] reported that lamellar CB was the most prevalent type (41.01%), followed by extensive (33.09%) and bulbous (25.89%) types. Our findings are consistent with this distribution, as the lamellar type was the most common on both sides in our study. In contrast, other studies involving adult populations have reported extensive [45] or bulbous [46] types as the most frequent. These discrepancies may be attributed to differences in imaging modalities and, more importantly, the age ranges of the study populations. Recent pediatric imaging studies have shown that paranasal sinus development continues throughout childhood and adolescence and that sinus morphology, pneumatization, and volume may vary according to age [16,17,45,47]. Therefore, the pediatric and adolescent age range of the present study should be considered when interpreting the prevalence and morphology of CB subtypes.

Regarding the lateralization of CB, Kalaiarasi et al. [48] and other researchers [49] observed a higher frequency on the right side. Our study mirrors this trend, with 100 right-sided versus 93 left-sided cases. Conversely, a large-scale study of 774 patients reported a left-sided predominance (49.48%) [50]. Such conflicting results in the literature may arise not only from variations in sample size and imaging techniques but also from potential ethnic and genetic differences in the sinonasal anatomy of the studied populations.

The reported prevalence of HC in the literature exhibits a wide range, from 2% to 43.9%, depending on the imaging modality, population characteristics, and diagnostic criteria used [8,18,51]. In our cohort, HC was identified in 47 patients on the right and 50 patients on the left, corresponding to prevalence rates of 28.1% and 29.9%, respectively, which align with the reported range in the literature. While the CBCT-based study by Pekiner et al. [52] reported a slight left-sided predominance (*n* = 47 vs. *n* = 36), our findings showed a nearly symmetrical distribution with only a minimal numerical difference. This suggests that HC development may not be strictly lateralized in the pediatric population.

However, in another CBCT-based study, the prevalence of HC was found to be higher on the right side (18.8%) compared with the left (11.8%) [53]. This discrepancy in lateralization across different studies may be attributed to variations in age range, interobserver variability in identifying smaller cells, and differences in sample sizes.

The causal relationship between NSD and CB development remains a subject of debate in the literature. El-Taher et al. [34] suggested that while NSD may not directly trigger CB formation, existing CB might increase the degree of pneumatization. In contrast, Sancar et al. [54] found no significant association. Our findings showed no statistically significant association between NSD severity and the presence of CB on either side (*p* > 0.05). Therefore, CB and NSD severity may not be developmentally linked in the present pediatric cohort. These conflicting results across studies, including the report by Erkan et al. [55] suggesting a mutual influence, highlight the need for larger, standardized longitudinal studies to clarify whether these variations are developmentally linked or purely coincidental.

Regarding the morphological types of CB, we found no significant association with NSD severity. This differs from the findings of El-Taher et al. [34], who reported a strong correlation, with 80% of severe NSD cases presenting with bulbous or extensive CB. This discrepancy likely stems from population differences; while our study cohort was a general pediatric/adolescent group, the aforementioned study consisted of symptomatic patients undergoing surgical treatment. It is plausible that in symptomatic individuals, the degree of anatomical variation is more pronounced, leading to more significant correlations between structural deformities.

While Atsal et al. [5] observed a significant increase in HC prevalence corresponding with the degree of NSD in an adult population (*p* < 0.001), our study found no significant association between NSD severity and HC presence on either side (*p* > 0.05). Beyond the larger sample size in the previous study, the age difference is a critical factor. The ethmoid air cell system, from which HC originates, undergoes significant expansion during puberty. Our pediatric focus may capture these variations at an earlier developmental stage before the potential compensatory relationship between septal deviation and accessory cell pneumatization becomes statistically evident.

In the present study, DMFT scores did not significantly differ according to the presence of CB or HC on either side (*p* > 0.05). Although slight numerical differences were observed between patients with and without these variations, these differences were inconsistent in direction and did not reach statistical significance. Therefore, CB and HC alone do not appear to be directly associated with caries experience in this pediatric cohort. In a study evaluating the presence of anatomical variations such as CB and HC in children with mouth breathing, CB was more frequently observed in children with mouth breathing, whereas the prevalence of HC did not differ significantly [56]. These findings suggest that nasal cavity and osteomeatal complex variations may exert indirect effects on oral health, highlighting the need for studies with larger sample sizes and multicenter designs.

Recent imaging-based studies have emphasized that paranasal sinuses and sinonasal structures continue to develop throughout childhood and adolescence, with age-related changes in pneumatization, volume, and morphology [16,17]. In addition, pediatric CBCT studies have reported that anatomical variations in the paranasal sinuses and nasal cavity, including NSD, CB, and HC, may be frequently observed in children and patients in the developmental age [18,19]. These findings support the need to consider the pediatric age range when interpreting the prevalence and morphology of sinonasal anatomical variations.

In our study, no statistically significant differences were identified between CB types and DMFT scores on either side. Among right-sided CB types, the highest mean DMFT score was observed in the bulbous type, and the lowest in the extensive type. On the left side, the lowest DMFT score was found in the extensive type, whereas the highest was in the group without CB. However, these numerical differences did not reach statistical significance, suggesting that CB types were not significantly associated with DMFT scores in the present pediatric cohort. These findings should also be interpreted in light of the pediatric age range of the present cohort, as sinonasal morphology may continue to change during childhood and adolescence.

In our study, a significant positive trend was identified using the Jonckheere-Terpstra test (*p* = 0.004). This indicates that as the severity of NSD increases (from Type I to Type III), DMFT scores also tend to increase. This finding supports the hypothesis that a higher degree of anatomical obstruction may be associated with more pronounced mouth breathing, which could contribute to increased caries risk. To the best of our knowledge, no study in the literature has specifically examined the effect of NSD severity on DMFT; however, studies assessing the relationship between mouth breathing and DMFT do exist. In a study by Ballikaya et al. [23] evaluating factors influencing mouth breathing syndrome in children, it was determined that children who breathed through the mouth had significantly higher DMFT scores compared to those who breathed through the nose. Similarly, Kimura et al. [9] concluded that in children and adolescents, mouth breathing may be associated with dental caries due to changes in salivary pH and oral microbiota. Since NSD may contribute to nasal airway obstruction and compensatory mouth breathing, it may be hypothesized that increasing NSD severity could be associated with higher DMFT scores. However, mouth-breathing status, nasal airflow, salivary flow, oral pH, and oral microbiota were not directly evaluated in the present study. Therefore, this proposed pathway represents a hypothetical biological explanation rather than a mechanism demonstrated by the collected data.

From a clinical perspective, these findings may suggest that children with more severe NSD detected on CBCT could benefit from closer oral health monitoring and, when clinically indicated, otorhinolaryngological evaluation. However, severe NSD should not be considered an independent predictor of caries experience, but rather a possible radiological finding that may support multidisciplinary assessment in selected pediatric patients. In addition, the number of patients with severe NSD, Type III, was relatively small. Therefore, the statistical trend across NSD severity categories should be considered an associative finding rather than strong evidence of a robust dose-dependent relationship.

The results of our study’s multiple linear regression analysis revealed that both age and NSD severity were significantly associated with DMFT scores (β = 0.206, *p* = 0.005). This finding indicates an association between greater NSD severity and higher DMFT scores. Although nasal obstruction, compensatory mouth breathing, and reduced salivary protection may represent possible biological explanations, these parameters were not directly assessed in the present study and the underlying mechanism cannot be determined from the available data. Although higher DMFT scores have been reported in children who breathe through their mouths in the literature [23], our study contributes to the literature by evaluating this relationship in relation to the radiologically assessed severity of NSD. These findings suggest that NSD severity may be associated with DMFT scores independently of age and sex within the limitations of the present retrospective radiological dataset. Sex was not significantly associated with DMFT scores in the regression model, suggesting that the observed association between NSD severity and DMFT was similar in males and females within this study population. However, the present data do not allow conclusions regarding potential sex-related differences in the underlying biological mechanisms.

The regression model established in our study explains 14.9% of the variance in DMFT. This finding indicates that other variables not included in the regression model, such as dietary habits, oral hygiene routines, and socioeconomic status, may also substantially contribute to DMFT scores. Since these variables were not incorporated into the present analysis, it remains unclear to what extent the observed relationship may be influenced by these unmeasured factors. Therefore, the findings do not allow for the determination of a fully independent effect of NSD on caries experience.

Nevertheless, the presence of a consistent trend across increasing NSD severity levels may indicate a possible contributory role, which should be further explored in studies with more comprehensive variable control.

Our study has several limitations. Due to its retrospective design, only associations could be evaluated, and causal inferences cannot be established. The DMFT index was assessed solely based on radiological data obtained from CBCT images, without intraoral clinical examination. Since the DMFT index was calculated only for permanent teeth, caries experience in primary teeth was not included, which may have led to an underestimation of the total caries burden in younger children. In addition, the radiological assessment had limited sensitivity for identifying early-stage, non-cavitated enamel lesions. Therefore, initial caries lesions may have been systematically underdiagnosed, potentially leading to an underestimation of the true caries burden. This limitation is particularly relevant in pediatric populations, where early enamel lesions are more prevalent. Consequently, this underestimation may have attenuated the strength of the associations observed in this study, particularly in relation to NSD severity.

In addition, several important individual, behavioral, and environmental factors known to influence DMFT scores—such as socioeconomic status, parental education, dietary habits, sugar consumption, oral hygiene practices, fluoride exposure, access to dental care, and systemic diseases [14,15]—were not included in the analysis. The absence of these potential confounding variables limits the ability to determine whether the observed association between NSD severity and DMFT scores is independent of these well-established caries-related factors. Therefore, the relationship observed in the present study should be interpreted cautiously and should not be considered evidence of an independent causal effect of NSD on caries experience.

Another limitation is the relatively small number of patients in the severe NSD group, Type III, which included only 11 patients. This may reduce the robustness of comparisons across NSD severity categories and should be considered when interpreting the statistically significant ordered trend between NSD severity and DMFT scores.

Furthermore, the relatively limited sample size and the single-center design may restrict the generalizability of the findings. Future large-scale, multicenter studies incorporating both clinical and radiological assessments, as well as comprehensive control of confounding variables, are warranted to validate and expand upon these results.

## 5. Conclusions

The observed association between NSD severity and DMFT scores suggests that severe NSD may be considered a potential radiological factor associated with higher caries experience in children. However, because of the retrospective design of the study and the radiological assessment of DMFT, causal conclusions cannot be drawn. In contrast, CB and HC were not significantly associated with DMFT scores. When CBCT scans are already available for pediatric dental indications, they may provide additional information on sinonasal anatomical variations. The potential relevance of severe NSD for multidisciplinary clinical assessment should be interpreted cautiously and confirmed in prospective studies incorporating comprehensive dental and otorhinolaryngological evaluations.

## Figures and Tables

**Table 1:** Prevalence of NSD severity and direction, presence and types of CB, and presence of HC.

		*n* (%)
**NSD Severity**	Type 1	106 (63.5)
	Type 2	50 (29.9)
	Type 3	11 (6.6)
**NSD Direction**	Left	83 (49.7)
	Right	84 (50.3)
**Right CB Types**	Bulbous	15 (9.0)
	Extensive	38 (22.8)
	Lamellar	47 (28.1)
	Absent	67 (40.1)
**Left CB Types**	Bulbous	10 (6.0)
	Extensive	40 (24.0)
	Lamellar	43 (25.7)
	Absent	74 (44.3)
**Right HC**	Present	47 (28.1)
	Absent	120 (71.9)
**Left HC**	Present	50 (29.9)
	Absent	117 (70.1)

NSD = Nasal Septal Deviation, CB = Concha Bullosa, HC = Haller Cells.

**Table 2:** Relationship between NSD severity/direction and the presence of CB and HC on the right and left sides.

		NSD Severity *n* (%)				NSD Direction *n* (%)		
		Type 1	Type 2	Type 3	*p*	Right	Left	*p*
**Right CB**	Present	62 (58.5)	31 (62.0)	7 (63.6)	0.885	54 (64.3)	46 (55.4)	0.243
	Absent	44 (41.5)	19 (38.0)	4 (36.4)		30 (35.7)	37 (44.6)	
**Left CB**	Present	60 (56.6)	25 (50.0)	8 (72.7)	0.370	44 (52.4)	49 (59.0)	0.387
	Absent	46 (43.4)	25 (50.0)	3 (27.3)		40 (47.6)	34 (41.0)	
**Right HC**	Present	27 (25.5)	18 (36.0)	2 (18.2)	0.295	21 (25.0)	26 (31.3)	0.363
	Absent	79 (74.5)	32 (64.0)	9 (81.8)		63 (75.0)	57 (68.7)	
**Left HC**	Present	29 (27.4)	17 (34.0)	4 (36.4)	0.623	27 (32.1)	23 (27.7)	0.532
	Absent	77 (72.6)	33 (66.0)	7 (63.6)		57 (67.9)	60 (72.3)	

Chi-square test, NSD = Nasal Septal Deviation, CB = Concha Bullosa, HC = Haller Cells.

**Table 3:** Relationship between the Presence of CB and HC and DMFT Scores.

		DMFT Mean ± SD	Median (Min–Max)	Mean Rank	U Value	*p*
**Right CB**	Present	2.78 ± 2.68	2 (0–13)	89.29	2821.000	0.079
	Absent	2.45 ± 3.22	2 (0–15)	76.10		
**Left CB**	Present	2.64 ± 2.55	2 (0–15)	87.53	3112.000	0.282
	Absent	2.66 ± 3.31	2 (0–13)	79.56		
**Right HC**	Present	2.51 ± 2.05	2 (0–9)	88.84	2592.500	0.411
	Absent	2.70 ± 3.18	2 (0–15)	82.10		
**Left HC**	Present	2.86 ± 2.89	2 (0–15)	89.74	2638.000	0.308
	Absent	2.56 ± 2.91	2 (0–13)	81.55		

Mann–Whitney U test, CB = Concha Bullosa, HC = Haller Cells, DMFT = Decayed, Missing, and Filled Teeth.

**Table 4:** Relationship between NSD Severity and DMFT Scores.

	Number of NSD Groups	N	Observed J−T	Standardized J−T (z)	*p*
DMFT	3	167	4377.50	2.893	0.004 *

* *p* < 0.05, Jonckheere-Terpstra test, NSD = Nasal Septal Deviation, DMFT = Decayed, Missing, and Filled Teeth.

**Table 5:** Multivariate Linear Regression Analysis for Factors Associated with DMFT.

	B	Standard Error	β	t	*p*	95% CI (Lower–Upper)
Constant	−2.535	1.216	-	−2.084	0.039	(−4.937, −0.133)
Age	0.323	0.076	0.308	4.253	0.001 *	(0.173, 0.474)
NSD severity	0.970	0.342	0.206	2.837	0.005 *	(0.295, 1.646)
Sex	−0.152	0.419	−0.026	−0.362	0.718	(−0.979, 0.675)

* *p* < 0.05, Model statistics: R = 0.386, R^2^ = 0.149, Adjusted R^2^ = 0.133, F = 9.517, *p* < 0.001, DMFT: Decayed, Missing, and Filled Teeth; NSD: Nasal Septal Deviation. NSD severity was entered as an ordinal variable. Sex was coded as 0 = female and 1 = male.

## Data Availability

The datasets analyzed during the current study are available from the corresponding author upon reasonable request.
